# A mixed methods study to understand perinatal mental healthcare referral decisions among midwives and health visitors in the UK

**DOI:** 10.3389/fpsyt.2023.1056987

**Published:** 2023-06-12

**Authors:** Joanne Johnson, Lucy Hope, Lisa Jones, Eleanor Bradley

**Affiliations:** ^1^Herefordshire and Worcestershire Health and Care NHS Trust, Worcestershire, United Kingdom; ^2^College of Health, Life and Environmental Sciences, University of Worcester, Worcester, United Kingdom; ^3^Department of Psychological Medicine, University of Worcester, Worcester, United Kingdom

**Keywords:** referral decision making, perinatal mental health, midwifery, health visiting, referral and consulation, surveys and questionnaires, mixed methods

## Abstract

**Background:**

In the UK approximately half of women requiring perinatal mental health (PNMH) care do not receive treatment despite having routine contact with midwives (MWs) and health visitors (HVs). Limited research has been undertaken regarding MWs’/HVs’ decision-making around referring women for secondary PNMH care. In particular, the impact that the level of local secondary PNMH services may have on MWs’/HVs’ referral decisions is unexplored.

**Aim:**

To understand MWs’/HVs’ decision-making in relation to referring women with identified PNMH problems, to identify barriers and facilitators to effective and timely referrals including any impact of the local secondary PNMH service provision.

**Methods:**

Participants were recruited from four National Health Service (NHS) Trusts in England, located across two geographical areas, that provided different types of PNMH services. One area had PNMH services that met National Institute for Health and Care Excellence (NICE) guidelines; the other area had no secondary PNMH services. A sequential mixed methods design was used: In-depth semi-structured interviews with practising MWs/HVs (*n* = 24) to explore their approach to PNMH referral decision-making, analysed using thematic analysis; Questionnaire offered to all practising MWs/HVs in the two geographical areas to measure factors that may impact on PNMH referral decision-making allowing for statistical comparisons to be made between the professional groups/geographical areas.

**Findings:**

Three themes were identified from the interviews that impacted on MWs’/HVs’ PNMH referral decision-making: identifying need; education, skills and experience; and referral pathways.

Questionnaire response rate 13.1% (*n* = 99). The most reported facilitators to referral decision-making were a trusted relationship between MWs/HVs and women and routine enquiry about women’s mental health; the most reported barriers were stigma associated with mental ill-health and women’s perceived fear of child removal.

**Conclusion:**

Fundamental to MWs’/HVs’ decision-making was their perceived relationship between themselves and women. Although PNMH service provision is important for women to ensure they receive appropriate PNMH care, service provision appeared less important to MWs’/HVs’ referral decision-making than how maternity/health visiting services were delivered. Further important factors to MWs/HVs were to the ability to provide continuity of carer with women allowing MWs/HVs to identify women who would benefit from referral for secondary PNMH care.

## Introduction

Perinatal mental health (PNMH) is a major public health concern, both in the UK (1) and internationally (2). PNMH disorders include new-onset conditions occurring during pregnancy and after childbirth and pre-existing conditions that relapse or recur such as antenatal and postnatal depression, anxiety disorders, posttraumatic stress disorder (PTSD), obsessive compulsive disorder (OCD), tocophobia, schizophrenia, bipolar disorder and postpartum psychosis (PP) (3).

Depression and anxiety disorders are the most prevalent health problems in the perinatal period (4) with up to 20% of women reported to experience a depressive episode (5) and an estimated 22% of women affected by perinatal anxiety (6). Left untreated, PNMH problems can have adverse consequences for both mother (7) and child (8) including but not limited to poor obstetric outcomes and suicidal ideation/attempt, and the potential for parents not being able to care for their infant, leading to abuse and neglect. However, appropriate interventions can prevent or reverse the negative trajectory of maternal depression (9) where early identification and intervention in PNMH disorders can improve outcomes for mother and child (10).

The perinatal period provides healthcare professionals (HCPs) with the opportunity to assess a woman’s emotional wellbeing and risk of PNMH disorders due to the frequency of contacts during this time (4, 11). In the UK, midwives (MWs) and health visitors (HVs) meet with women regularly guided by NICE (12, 13) and Healthy Child Programme (14) and play a key role in assessing and referring to secondary PNMH care (11) due to the routine nature of contact offered in the perinatal period. Countries outside the UK also recognise the importance of routine screening and assessment of women’s PNMH. For example, Australia recommends that appropriately trained HCPs [including GPs, MWs, maternal and child health nurses (HVs) and obstetricians] complete PNMH screening in the antenatal period, 6–12 weeks after birth and at least once in the first postnatal year using validated screening tools (15) mirrored by those recommended by NICE (12). Similarly, France has a well-established PNMH infrastructure that recommends early PNMH screening and provides both in-patient and out-patient care for women akin to UK (16). However, despite routine contact with MWs and HVs, an estimated 50% of PNMH UK cases go undetected and untreated (17). Of those that are detected, 40% receive appropriate treatment (18). Research suggests MWs and HVs may lack knowledge and confidence in recognising and managing mental ill-health and lack expertise in screening for PNMH disorders and referring women in need of PNMH care (19–22). This may partially explain why many women with PNMH problems go undetected or fail to receive appropriate treatment.

UK NICE (11) guidance recommends clinical organisations provide specialist multidisciplinary perinatal services in each locality with clear referral and management protocols for services. Women requiring PNMH care should have access to care and treatment from specialist PNMH Community Mental Health Teams (CMHTs), specialist Mother and Baby Units (MBUs), specialist PNMH MWs and specialist PNMH HVs (3, 11, 22–24). However, UK service provision for PNMH is variable in both coverage (25) and quality (26) and an estimated 40% of women in England do not have access to specialist PNMH service provision (18).

Research also demonstrates that in addition to a lack of PNMH service provision, HCPs including MWs and HVs lack policy direction and referral criteria to guide practice when managing women with recognised PNMH problems (19, 20, 26). Furthermore, there is a dearth of literature concerning what influence having a specialist secondary PNMH service has on MWs’ and HVs’ decision-making when referring women for PNMH care. The term ‘secondary care’ denotes PNMH care services provided by experts outside of primary care (GP, midwife, health visitor). Women access secondary care through a healthcare professional referral not self-referral. Specialist care denotes PNMH care provided by a healthcare professional, for example, a midwife having undertaken specific additional specialist PNMH training and operates within a multidisciplinary team. As mental ill-health is the most common perinatal illness and that an estimated 50% of PNMH cases go undetected, the following research question and overarching aims were developed:

What factors influence midwives’ and health visitors’ decision-making in relation to referring women for secondary perinatal mental health care?

## Aims

To understand MWs’/HVs’ decision-making in relation to referring women with identified PNMH problems, to identify barriers and facilitators to effective and timely referrals including any impact of the local secondary PNMH service provision.

## Methods

This study utilised a sequential mixed methods design delivered in two phases. Sequential exploratory design involves collecting data following an iterative process where the data collected in one phase contributes to the data collected in subsequent phases (27). The first phase involved semi-structured interviews with practising MWs and HVs from four participating NHS Trusts across England. These Trusts covered two geographical areas. Area 1 included three NHS Trusts that provided NICE recommended PNMH services (11); Area 2 included one NHS Trust that, at the inception of the study, had no secondary PNMH services (during Phase 2 of the research, Area 2 acquired a secondary PNMH CMHT that was in place for less than 12 months at completion of Phase 2). In Phase 1 an interview guide (Supplementary material) was developed to address the aims of the research and posed questions about professional decision-making around PNMH care and explored any impact of having a local secondary PNMH service on MWs’ and HVs’ approach to PNMH referral decision-making. The interview guide was modified following review by a team of expert academics in perinatal psychiatry, psychology and midwifery, and practising health care professionals. Phase 1 interviews were conducted face-to-face or *via* telephone and ranged from 14 min to 1 h and 21 min, with a mean duration of 36 min. The interview findings were used to inform the development of the questionnaire used in Phase 2.

Phase 2 involved a cross-sectional survey design implemented through a bespoke anonymised questionnaire, including closed and open questions in four sections: demographic information; identifying PNMH need; education, skills and experience; and referral pathways based on the themes developed in Phase 1. For closed questions a five-point Likert scale was used (1 = not a major barrier/facilitator to 5 = a major barrier/facilitator). A 6th option of unsure/not applicable was also added. The questionnaire underwent piloting with academics in psychology and midwifery, PhD students, practising MWs and HVs, experts in perinatal psychiatry and experienced researchers where feedback was given on the quality and clarity of questions and online functionality before finalising the questionnaire.

Ethics approval was granted from the University of Worcester Health, Life & Environmental Sciences Research Ethics Panel (SH17180018-R) and approval to conduct the research in the NHS from the Health Research Authority (HRA) (235568). Participant information sheets were given to all prospective participants and consent was given by participants in each phase of the study.

### Participants

All practising MWs and HVs employed by the four participating NHS Trusts were invited to participate in each phase of this study. Twenty-four participants were recruited into Phase 1 (MWs = 16; HVs = 8; Area 1 = 15; Area 2 = 9) from May 2018 to October 2018. Invitations to participate in each phase of the study were sent by email with one reminder 2 weeks after the initial invitation. The questionnaire was open from January 2020 to March 2020. Ninety-nine responses were received from a sampling population of 755 MWs and HVs from the four participating Trusts (response rate 13.1%). A breakdown of the number of responses and response rate by professional group and geographical area is shown in Table 1.

**Table 1:** Number of respondents and response rate by area and professional group for phase 2.

Geographical Area	MW or HV	Number of Respondents	Number of Respondents	% Response	Total n (%) Response by Area
Area 1	MW	457	38	8.32	56/642 (8.72)
	HV	185	18	9.73	
Area 2	MW	82	22	26.8	43/113 (38.0)
	HV	31	21	26.8	

### Data analysis: Phase 1

Qualitative data was analysed using Thematic Analysis (TA) based on Braun and Clarkes seven-step process (28). Interviews were transcribed verbatim by JJ and identifiable details removed. Transcripts were read and re-read to begin immersion in the data. The organic process of generating codes was achieved by examining transcripts line by line and highlighting repeated words and concepts to identify semantic and latent codes relating to the research aims. Emerging themes generated from TA were regularly reviewed and refined with EB until agreement was reached to establish trustworthiness of the data. This reflexive approach was employed to minimise bias owing to professional or personal experience, supporting an inductive rather than deductive approach (see Table 2).

**Table 2:** Themes and sub-themes generated from thematic analysis of interview data.

**Identifying need**
Continuity of carer
Disclosure
Time
**Education, skills and experience**
Targeting resources
Intuition and confidence
**Referral pathway**
Use of screening tools
Knowledge of referral pathway

### Data analysis: Phase 2

Quantitative questionnaire data were analysed using the SPSS Statistics version 26. Due to the modest sample size and small number of responses for each response option when stratified by geographical area and professional group, Likert item variables were collapsed into two options (not a major barrier/facilitator versus a major barrier/facilitator). Response frequencies were compared between professional groups and geographical areas using chi-squared tests or Fisher’s exact tests (FET). *p-values* ≤ 0.05 were considered statistically significant. Qualitative data from the open text responses were analysed broadly based on the concept of content analysis (29).

## Results

### Phase 1 results

**Figure 1:**
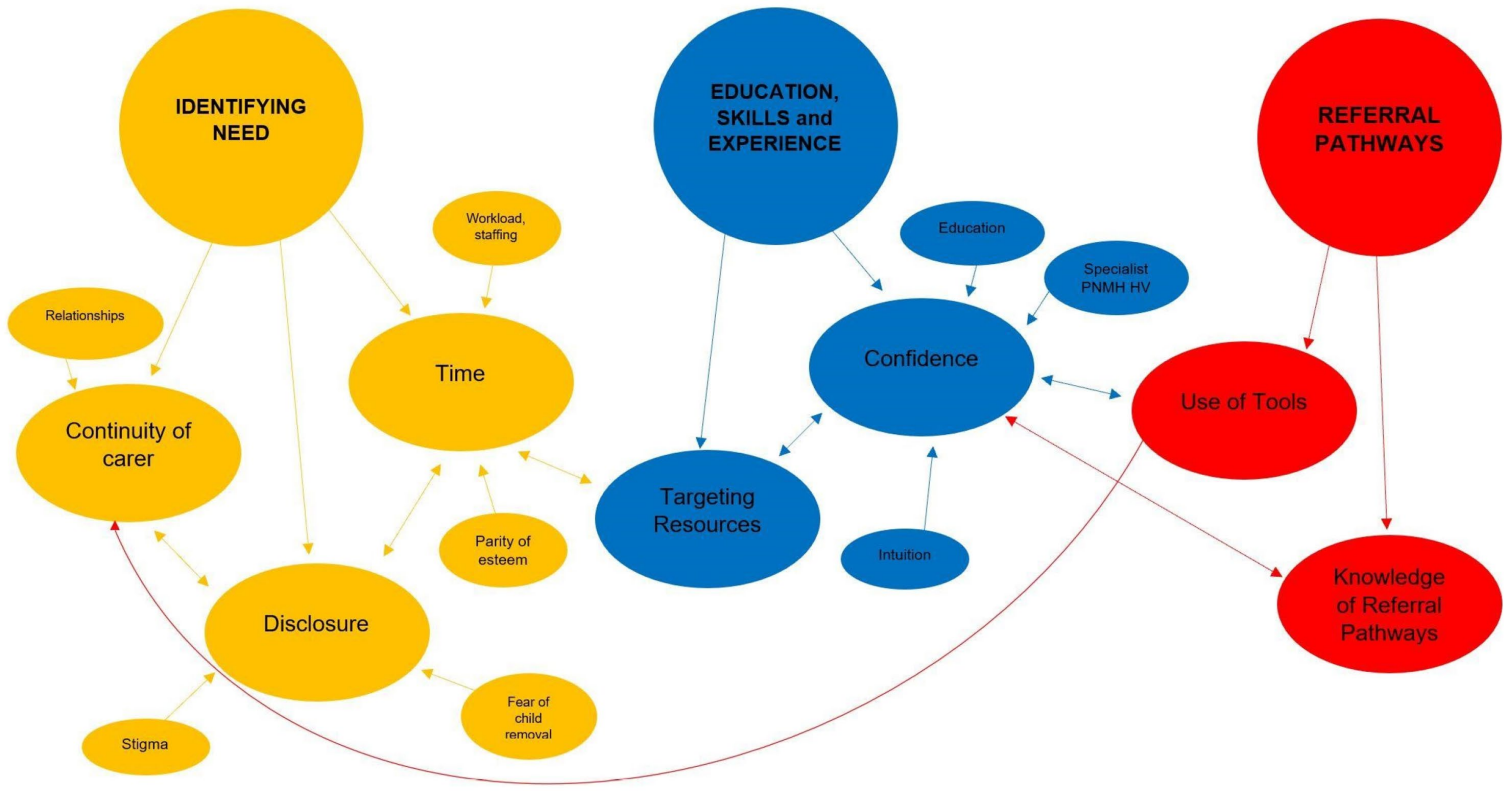
Mapping of themes and subthemes.

Three main themes were generated from the data: Identifying need; education, skills and experience; and referral pathways. The themes illustrate the barriers and facilitators described by the participants around their decision-making on referring women for PNMH care. Findings are presented under theme headings and sub-headings (Figure 1) with extracts from the transcripts to illustrate the data (further extracts in Supplementary material). Extracts have a unique identifier to indicate the participants profession and colour coded according to area (Blue to indicate Area 1 and Black to indicate Area 2 participants).

Identifying need: Participants discussed that when referring women for PNMH care they must first identify women in need of PNMH care. Certain factors impacted on their ability to identify this need, one of which was the presence or absence of continuity of carer (CoC).

#### Continuity of carer

Both groups of participants appeared to view CoC positively and perceived it as important when identifying PNMH problems. For example, when asked to identify supportive measures for perinatal mental health assessment respondents answered:

“*I think first of all you’ve got to gain a better, you know, build a relationship with the woman. I think continuity helps as well and good communication*…” (MW6)

In addition to building relationships, CoC was also perceived to be important for the opportunity it gave professionals to monitor a woman’s mood and detect deterioration in mood over time. Some HVs discussed the lack of continuity where community nursery nurses (CNNs) were carrying out contacts that were previously carried out by HVs. This was perceived to be a missed opportunity for HVs to identify any PNMH problems and demonstrated the changes in health-visiting services and the potential impact that the lack of CoC may have on identifying PNMH problems.

HVs from both areas reported having fewer contacts with women in their care which meant fewer opportunities to build a relationship with women and for women to disclose mental health problems.

#### Disclosure

For many participants across both professional groups and geographical areas, identifying women’s mental health problems were dependent on whether women disclosed a history of and/or current PNMH problems when asked. Some respondents challenged the belief that a trusted relationship between professional and woman resulted in disclosure of PNMH problems and believed that disclosure was dependent on women’s willingness to share information:

“*I’m a firm believer that clients tell you what they want you to hear, no matter how open* (you are)*, how ‘You can tell me anything’ you are, you are going to get those who feel that they can’t say that* (disclose PNMH problems) to you.” (HV4)

However, 16/24 (67%) participants perceived disclosure was facilitated by the relationship between professionals and women.

There was an acknowledgement amongst most of the participants from both geographical areas that the perceived stigma associated with mental health amongst women was a barrier to disclosure:

“…*People thinking mental health is still something to be ashamed of. I’d say that’s the biggest barrier.*” (HV8)

In addition to discussing strategies that encouraged disclosure, some participants also discussed a barrier to disclosure. MWs and HVs identified that some women feared disclosing PNMH concerns would result in removal of their child. This perception of fear posed a significant barrier to disclosure:

“*People don’t want to admit to their friends and family and also some appear less likely to admit* (PNMH problems) *to professionals as well. Erm* … *fear of you know, what the referral pathway is, you know, the classic* ‘*Oh are my children going to be taken off me?*’” (MW13)

#### Time

For most of the participants time, or rather the lack of time, was a barrier to identifying women with a history of and/or current PNMH problems. Participants cited issues such as workload, staffing levels and organisational changes in services that governed the amount of time they had to effectively assess women for PNMH problems:

“*Time is the biggest killer for us really. We’re limited* … *we haven’t got sufficient staff to cope* … *so I can’t honestly say, I know hand on heart these women get enough time.*” (MW3)

Some participants also discussed time in terms of capacity and workload in general. As a result of the service changes, HVs reported managing demanding caseloads and highlighted the lack of capacity to carry out some mandated contacts such as the antenatal contact. Many MWs from both areas suggested that they had limited time with women to address any mental health issues due to the need to complete physiological assessments. The interview data suggested that parity of esteem, defined by the Royal College of Psychiatrists as valuing mental health equally with physical health (30), was not evident as physical health checks took priority during contacts between some MWs and women.

### Education, skills and experience

The theme of education, skills and experience related to the barriers and facilitators when identifying women with PNMH concerns and referring them for PNMH care and encompassed the skills involved in professional decision-making within MWs’ and HVs’ roles.

#### Targeting resources

Both geographical areas had undergone recent changes in service delivery. The sub-theme of ‘targeting resources’ captured how participants utilised their professional knowledge, judgment and skills to target the limited resources to women whom they perceived as needing PNMH care:

“…*Our services are very stretched. We’ve got a lot less health visitors. We can’t provide the service that we could historically* … *we try to target the ones that are most vulnerable.*” (HV1)

The interview data implied that despite the recommended contacts for women in the perinatal period, MWs and HVs from both areas perceived their services were “very stretched” (HV1) in terms of staff shortages and appeared dissatisfied with the service they were providing:

“*Well I think the most important thing is the decline in the service as erm*, … *the service provided to women with poor perinatal mental health. We used to provide a very good service with the listening visits. The tools we used worked well and since we’ve stopped using the EPDS and using the universal* (questions) *we don’t capture as many women* … *you know, a lot of women we’re missing* … *which will then have a knock-on effect with children’s health as well.*” (HV7)

#### Intuition and confidence

When considering women’s PNMH, intuition played an important part in participants’ decision-making in the assessment and referral processes. Although many participants used the recommended screening tools, e.g., Universal PNMH questions (11) to guide decision-making, the tools were only one component when deciding whether to make a referral.

Many participants discussed using their intuition based on previous experiences and knowledge when assessing women’s needs for PNMH care. The participants were an experienced group of professionals (ranging from 1 year to 33 years post qualification, mean length of qualification was 19 years) and the data suggested that individual practitioners drew on their experiences when managing women with PNMH problems and this appeared to give them confidence:

“*Yeah I do* (feel confident). *I do, but I think that’s because I’ve been nursing a long time. And erm, I worked at* [MBU] *Unit and so had a lot of support there* … *Erm I do feel confident really*…” (HV7)

Intuition and history taking appeared to be important factors for MWs and HVs to consider for women who presented as ‘well’ but were at a high risk of becoming unwell. Intuition was often described in less tangible terms whereby participants ‘had a feeling’ that something was wrong but had no concrete evidence to support this feeling:

“*So, you know,* … *your gut feeling and how you notice things like, I can remember* … *seeing a mom and a baby’s interaction and thinking that makes me feel stressed, what is going on there?*” (HV1)

Most participants from both geographical areas stated they lacked appropriate PNMH training and would like further education/training despite receiving regular mandatory updates on PNMH. The minimal training appeared to impact on a practitioner’s confidence when supporting women with PNMH problems:

“*Erm I think confidence is a difficult one to assess because we’ve identified the lack of training, you always feel you could and should be doing more. But you don’t know what the ‘more’ is or what it looks like.*” (MW11)

Some MWs, whilst they had access to mandatory updates/training on PNMH, were dissatisfied with this and how it was delivered:

“*It would be good if there was some sort of compulsory study day or interaction I think, to make mental health education within the profession more tangible and meaningful. I don’t think it’s good enough using e-learning personally.*” (MW13)

The interviews revealed that the MWs practicing in both geographical areas expressed a desire for PNMH training/education. This suggested that those MWs working in an area with specialist secondary PNMH service provision did not necessarily have increased knowledge and confidence when dealing with women’s PNMH needs:

“…(We have) *mandatory updates* (on PNMH) *but it’s limited. We would always look to* (specialist midwife) *for support because we haven’t got the knowledge really. We’ve got basic knowledge and signs to look for but apart from that, that’s it.*” (MW3)

There was a marked difference between the professions regarding desire for training/education in PNMH; 15 out of 16 MWs wanted more training in PNMH compared to only 3 out of 8 HVs. Importantly, one HV from Area 2 highlighted that PNMH education has its limitations if services were not available once PNMH problems have been identified:

“*We can identify all we like, we can have all the education we like but, in the end, we haven’t got anywhere to refer people to who have the expertise who can help them.*” (HV2).

The data suggested that of the two professions, HVs appeared more knowledgeable about PNMH than MWs, regardless of their geographical area.

### Referral pathways

The first two themes contextualised participants’ experiences of referring women for secondary PNMH care by offering insights into factors influencing their professional decision-making. The final theme addressed one of the aims of the research which was to explore any impact of having a local secondary PNMH service on MWs’ and HVs’ approach to decision-making when deciding whether to refer women for PNMH care. The sub-themes generated from the data explored participants’ experiences of using referral pathways in the different geographical areas, i.e., areas with and without secondary PNMH services and the referral options available to the MWs and HVs.

#### Use of screening tools

Prior to deciding to refer women for PNMH care, MWs and HVs are required to screen women for risk of PNMH concerns using validated tools such as the Whooley questions, GAD-2, PHQ-2 and the EPDS (11). During the interviews, participants were asked if they used a screening tool as an aid to assess women for PNMH problems. The data suggested a disconnect between NICE guidelines and practice regarding the use of screening tools and PNMH referrals. Approximately a quarter of all midwives interviewed were unaware of any screening tools for PNMH problems. Despite stating they were unaware of any screening tools, some MWs failed to recognise that the questions they were prompted to ask on their electronic records were screening tools. In contrast, all HVs interviewed were aware of screening tools.

A notable difference between areas was that following screening for PNMH problems, Area 2 participants had limited referral options and no option of referring women for specialist PNMH care. The quote by MW13 reveals an awareness of PNMH risk factors but hints at an element of uncertainty about where to refer women with PNMH problems with the limited options available:

“*So if there’s history* (of mental health), *so if she discloses* (PNMH problems) *and we have concerns, so if there’s any reports of self-harm erm or previous depression that was medicated or secondary care team involvement, that’s when we would think to do a referral* … *because there’s a risk factor there* … *it depends on the level of care we feel that woman would need, erm we’d refer to the GP, erm or the crisis team. It really would depend on the level of need*…” (MW13)

There was no consensus amongst the HVs across both geographical areas regarding the value of screening tools. Participants identified the Universal questions as more effective if HVs visited the women more and built a relationship that would encourage disclosure during screening.

Interview data suggested that organisational changes in health-visiting services resulted in fewer contacts and led some participants (HV6) to question the effectiveness of the Universal PNMH questions as a result. Some participants believed the success of the Universal PNMH questions was determined by the accompanying conversation a practitioner had regarding mental health and well-being. In HV5’s opinion, it is important that HVs have a conversation about mental health before asking the questions; otherwise the questions are rendered useless:

“*It’s not just about asking those four questions, it’s about how they* (the HVs) *set their stall out. So, it’s the conversation they have about mental health and well-being before they ever ask those questions. It’s about being very clear about what it is you are asking* … *For me, you know, the biggest component for that universal assessment is the discussion you have about mental health and well-being and it’s about setting your stall out* … *Just going into somebody and just launching into ‘During the past month have you* …*.’ You might as well not bother.*” (HV5)

### Knowledge of referral pathways

Referral pathways (sometimes referred to as care pathways) ensure all primary and secondary HCPs know how to access assessment, referral and treatment options for pregnant and postnatal women (11). Secondary services, both specialist PNMH services and general mental health services, often place specific eligibility criteria on referrals. However, some participants stated finding their respective Trust referral pathways unclear and difficult to navigate. This presented a barrier when deciding to refer women for secondary PNMH care.

In addition to unclear pathways, participants in Area 2 highlighted the lack of PNMH services and demonstrated confusion about where to refer women and frustration due to lack of services:

“*I’d say it’s availability of services definitely* (that’s a barrier to referring] *because there is a certain point that you sometimes think ‘Oh my God, what can I do?’, you know? You know we really want you to tell us* [about your mental health problems) *and we really want to be able to deal with these sorts of things and there’s just nothing to offer you* (in terms of services)…” (HV2).

“*I think what you need to take away from this* (interview) *is it is fine* (that mental health is being recognised) *but if there is no support in the background, it’s just a nice conversation* … *So I think we just need a bit more robust support, but that would require finances and support*…” (MW15).

According to the data HVs appeared more familiar with PNMH screening tools than MWs. The data indicated a lack of knowledge and confidence in using screening tools which suggests some participants would benefit from training/education in using tools. Irrespective of geographical area, many of the MWs and HVs perceived their referral pathways were unclear. Most professionals from Area 2 (8 out of 9 participants interviewed in Phase 1) wanted a clearer referral pathway and dedicated PNMH services. Interview data indicated that some participants from Area 2 felt frustrated and helpless due to the lack of secondary PNMH services in their area which consequently meant that women did not receive the specialist support and care they required.

### Phase 2 results

A summary of the demographic characteristics of respondents is presented in Table 3. The majority of MWs (*n* 47) were hospital based and all HVs (*n* 39) were based in the community.

**Table 3:** Comparisons of response rates by base of work.

Base of work	Area 1		Area 2		*X*^2^ (*p*-value)	MW *n* 60 (%)	HV *n* 39 (%)	*X*^2^ (*p*-value)	Area 1 *n* 56 (%)	Area 2 *n* 43 (%)	*X*^2^ (*p*-value)
	MW *n* 38 (%)	HV *n* 18 (%)	MW *n* 22 (%)	HV *n* 21 (%)							
Hospital	30 (78.9)	0 (0)	17 (78.9)	0 (0)	83.931**(0.000)**	47 (78.3)	0 (0)	83.805**(0.000)**	30 (53.6)	17 (39.5)	3.152 (0.207)
Community	2 (5.3)	18 (100)	2 (9.1)	21 (100)		4 (6.7)	39 (100)		20 (35.7)	23 (53.5)	
Both	6 (15.8)	0 (0)	3 (13.6)	0 (0)		9 (15)	0 (0)		6 (10.7)	3 (7.0)	

*n*, number; (), % of respondents; bold type, statistically significant result.

#### Potential barriers/facilitators to identifying PNMH problems

Table 4 shows that HVs from both areas were significantly more likely to perceive reduced number of contacts with women as a major barrier to identifying PNMH needs of women than their midwifery counterparts (82.1% of HVs compared to 43.5% of MWs). Significantly more HVs perceived lack of contacts in the home as a major barrier to identifying PNMH needs (71.8%) than MWs (48.3%). In line with the interview data, MWs were more likely to perceive physical health care being prioritised during contact time with women as a major barrier to identifying the PNMH needs of women (45.0%) compared to HVs (15.4%).

**Table 4:** Potential barriers to identifying PNMH needs of women by area and professional group.

How much of a potential barrier do you consider the following to you being able to identify PNMH needs of women in your care/on your caseload?		Area 1		Area 2		*X*^2^ (*p*-value)	MW *n* 60 (%)	HV *n* 39 (%)	*X*^2^ (*p*-value)	Area 1 *n* 56 (%)	Area 2 *n* 43 (%)	*X*^2^ (*p*-value)
		MW *n* 38 (%)	HV *n* 18 (%)	MW *n* 22 (%)	HV *n* 21 (%)							
Reduced number of contacts with women	**0**	19 (50.0)	4 (22.2)	15 (68.2)	3 (14.3)	16.754**(0.001)**	34 (56.7)	7 (17.9)	14.604**(0.000)**	23 (41.1)	18 (41.9)	0.006 (0.937)
	**1**	19 (50.0)	14 (77.8)	7 (31.8)	18 (85.7)		26 (43.3)	32 (82.1)		33 (58.9)	25 (58.1)	
Lack of contacts in the home environment	**0**	20 (52.6)	5 (27.8)	11 (50.0)	6 (28.6)	5.368 (0.147)	31 (51.7)	11 (28.2)	5.326**(0.021)**	25 (44.6)	17.(39.5)	260 (0.610)
	**1**	18 (47.4)	13 (72.2)	11 (50)	15 (71.4)		29 (48.3)	28 (71.8)		31.(55.4)	26 (60.5)	
Delegating contacts with women to other staff, e.g., CNN, MSW, etc.	**0**	24 (63.2)	7 (38.9)	16 (72.7)	12 (57.1)	5.033 (0.169)	40 (66.7)	19 (48.7)	3.162 (0.075)	31 (55.4)	28 (65.1)	0.962 (0.327)
	**1**	14 (36.8)	11 (61.1)	6 (27.3)	9 (42.9)		20 (33.3)	20 (51.3)		25 (44.6)	15 (34.9)	
Having generic clinics, e.g., booking, PN, 6–8 week clinics, etc. instead of personally seeing women in your care/on your caseload	**0**	20 (52.6)	8 (44.4)	13 (59.1)	6 (28.6)	4.671 (0.198)	33 (55.0)	14 (35.9)	3.459 (0.063)	28 (50.0)	19 (44.2)	0.330 (0.566)
	**1**	18 (47.4)	10 (55.6)	9 (40.9)	15 (71.4)		27 (45.0)	25 (64.1)		28 (50.0)	24 (55.8)	
Physical health checks/tasks taking up contact time with women allowing little or no time to conduct a mental health assessment	**0**	20 (52.6)	14 (77.8)	7 (31.8)	19 (90.5)	18.218**(0.000)**	27 (55.0)	33 (84.6)	15.537**(0.000)**	34 (60.7)	26 (60.5)	0.001 (0.980)
	**1**	18 (47.4)	4 (22.2)	15 (68.2)	2 (9.5)		33 (45.0)	6 (15.4)		22 (39.3)	17 (39.5)	
Lack of confidence in your ability to identify key risk factors for women at high risk of developing PNMH difficulties	**0**	32 (84.2)	16 (88.9)	18 (81.8)	19 (90.5)	0.886 (0.829)	50 (83.3)	35 (89.7)	(0.556)*	48 (85.7)	37 (86.0)	0.002 (0.962)
	**1**	6 (15.8)	2 (11.1)	4 (18.2)	2 (9.5)		10 (16.7)	4 (10.3)		8 (14.3)	6 (14.0)	
Lack of confidence in your ability to identify women who are experiencing PNMH health difficulties	**0**	32 (84.2)	17 (94.4)	18 (81.8)	19 (90.5)	1.873 (0.599)	50 (83.3)	36 (92.3)	1.669 (0.237)*	49 (87.5)	37 (86.0)	0.045 (0.832)
	**1**	6 (15.8)	1 (5.6)	4 (18.2)	2 (9.5)		10 (16.7)	3 (7.7)		7 (12.5)	6 (14.0)	

0, not a major barrier; 1, a major barrier; (), %of respondents; CNN, community nursery nurse; MSW, maternity support worker; PNMH: perinatal mental health; *, Fisher’s exact test; bold type, statistically significant results.

The majority of respondents reported relying on intuition was a major facilitator to them in identifying women experiencing PNMH problems but there was no statistically significant difference between MWs and HVs overall or between areas (Table 5). Relying on prior experience of identifying PNMH issues was reported to be a major facilitator when identifying women in need of PNMH care by a significantly greater proportion of HVs compared to MWs (92.3% of HVs, vs. 70.0% of MWs).

**Table 5:** Potential facilitators to identifying PNMH needs in women by area and professional group.

How important do you consider the following potential facilitators to identifying PNMH needs in women in your care/on your caseload?		Area 1		Area 2		*X*^2^ (*p*-value)	MW *n* 60 (%)	HV *n* 39 (%)	*X*^2^ (*p*-value)	Area 1 *n* 56 (%)	Area 2 *n* 43 (%)	*X*^2^ (*p*-value)
		MW *n* 38 (%)	HV *n* 18 (%)	MW *n* 22 (%)	HV n21 (%)							
Relying on your gut instinct/intuition to identify women who are experiencing PNMH difficulties	0	6 (15.8)	4 (22.2)	3 (13.6)	3 (14.3)	0.65 (0.885)	9 (15.0)	7 (17.9)	0.152 (0.697)	10 (17.9)	6 (14.0)	0.274 (0.601)
	1	32 (84.2)	14 (77.8)	19 (86.4)	18 (85.7)	51 (85.0)	32 (82.1)	46 (82.1)		37 (86.0)		
Relying on your prior experience of PNMH issues/difficulties/needs	0	11 (28.9)	2 (11.1)	7 (31.8)	1 (4.8)	7.34 (0.062)	18 (30.0)	3 (7.7)	(0.011)*	13 (23.2)	8 (18.6)	0.309 (0.578)
	1	27 (71.1)	16 (88.9)	15 (68.2)	20 (95.2)	42 (70.0)	36 (92.3)	43 (76.8)		35 (81.4)		
Using an assessment tool to identify perinatal mental health difficulties	0	11 (28.9)	2 (11.1)	14 (63.6)	4 (19.0)	15.67 **(0.001)**	25 (41.7)	6 (15.4)	7.591**(0.006)**	13 (23.2)	18 (41.9)	3.932**(0.047)**
	1	27 (71.1)	16 (88.9)	8 (36.4)	17 (81.0)	35 (58.3)	33 (84.6)	43 (76.8)		25 (58.1)		

0, not a major facilitator; 1, a major facilitator; PNMH, perinatal mental health; (), % of respondents; *, Fisher’s exact test; bold type, statistically significant result.

Other important factors in identifying PNMH needs of women that were reported by respondents in the open text comments, not addressed by the closed questions in the survey, were the lack of privacy for confidential discussions with women without the presence of family members; communication and liaison between the multi-disciplinary team to enable information sharing and care planning; and MWs/HVs having the appropriate communication skills to ask women questions to facilitate disclosure of PNMH problems.

#### Barriers and facilitators to women disclosing perinatal mental health problems

The majority of respondents perceived that a trusted relationship between themselves and women was a major facilitator for disclosure of PNMH problems, with no differences found between MWs and HVs or between areas. Similarly, most respondents perceived that routine questioning about mental health facilitated disclosure, with slightly but significantly fewer MWs reporting this (see Table 6). Perceived facilitators generated from open text comments reiterated the importance and value of CoC and having private spaces for confidential conversations.

**Table 6:** Potential facilitators to women disclosing PNMH difficulties by area and professional group.

How important do you consider the following potential facilitators to women disclosing PNMH difficulties?		Area 1	Area 1	Area 2	Area 2	*X*^2^ (*p*-value)	MW *n* 60 (%)	HV *n* 39 (%)	*X*^2^ (*p*-value)	Area 1 *n* 56 (%)	Area 2 *n* 43 (%)	*X*^2^ (*p*-value)
A trusted relationship between the woman and MW/HV	**0**	2 (5.3)	0 (0.0)	0 (0.0)	1 (4.8)	2.109 (0.550)	2 (3.3)	1 (2.6)	(0.100)*	2 (3.6)	1 (2.3)	0.128
	**1**	36 (94.7)	18 (100)	22 (100)	20 (95.2)		58 (96.7)	38 (97.4)		54 (96.4)	42 (97.7)	(0.100)*
MW/HV routinely asking women about their mental health	**0**	1 (2.6)	1 (5.6)	6 (27.3)	1 (4.8)	11.467**(0.009)**	7 (11.7)	2 (5.1)	(0.476)*	2 (3.6)	7 (16.3)	4.753
	**1**	37 (97.4)	17 (94.4)	16 (72.7)	20 (95.2)		53 (88.3)	37 (94.9)		54 (96.4)	36 (83.7)	(0.038)*

0, not a major facilitator; 1, a major facilitator; PNMH, perinatal mental health; *, Fisher’s exact test; bold type = statistically significant result.

Table 7 shows that most respondents reported the perceived stigma associated with mental ill-health was a major barrier to women disclosing PNMH problems, there were no statistically significant differences between professional groups or by area. The majority of respondents, except HVs in Area 1, perceived women fearing having their child removed as a result of disclosing PNMH problems was a major barrier.

**Table 7:** Potential barriers to women disclosing PNMH difficulties by area and professional group.

How much of a barrier do you consider the following are to women when disclosing PNMH difficulties?		Area 1		Area 2		*X*^2^ (*p*-value)	MW *n* 60 (%)	HV *n* 39 (%)	*X*^2^ (*p*-value)	Area 1 *n* 56	Area 2 *n* 43 (%)	*X*^2^ (*p*-value)
		MW *n* 38 (%)	HV *n* 18 (%)	MW *n* 22 (%)	HV *n* 21 (%)							
Women fearing their child will be removed from them/their care, *n* (%)	**0**	4 (10.4)	9 (50.0)	5 (22.7)	1 (4.8)	15.853**(0.001)**	9 (15.0)	10 (25.6)	1.726 (0.189)	13 (23.2)	6 (14.0)	1.345 (0.246)
	**1**	34 (89.5)	9 (50.0)	17 (77.3)	20 (95.2)		51 (85.0)	29 (74.4)		43 (76.8)	37 (86.0)	
Perceived stigma associated with mental healthdifficulties, *n* (%)	**0**	8 (21.1)	4 (22.2)	3 (13.6)	2 (9.5)	1.782 (0.619)	11 (18.3)	6 (15.4)	0.144 (0.704)	12 (21.4)	5 (11.6)	1.643 (0.200)
	**1**	30 (78.9)	14 (77.8)	19 (86.4)	19 (90.5)	49 (81.7)	33 (84.6)	44 (78.6)		38 (88.4)		

0, not a major barrier; 1, a major barrier; PNMH, perinatal mental health; bold type, statistically significant result.

#### Education, skills and experience

Table 8 shows that significantly more HVs than MWs, and significantly more respondents in Area 1 than Area 2, felt that their training had equipped them “very well” to identify and refer women for PNMH care across a range of PNMH scenarios: women experiencing PNMH problems, women at high risk of developing PNMH problems, and women requiring referral to secondary PNMH services. Table 9 illustrates that significantly more respondents in Area 1 had received PNMH training from a PNMH specialist compared to Area 2. The majority of HVs had received PNMH training as part of their professional training compared to MWs and this was statistically significant.

**Table 8:** MWs and HVs (*n* = 97) perception of how well PNMH training/education equipped them to identify and refer women with PNMH problems by area and professional group.

How well has the training/education in PNMH		Area 1		Area 2		*X*^2^ (*p*-value)	MW *n* 58 (%)	HV *n* 39 (%)	*X*^2^ (*p*-value)	Area 1 *n* 55 (%)	Area 2 *n* 42 (%)	*X*^2^ (*p*-value)
		MW *n* 37 (%)	HV *n* 18 (%)	MW *n* 21 (%)	HV *n* 21 (%)							
Equipped you to identify women who are experiencing perinatal mental health difficulties?	**1**	3 (7.9)	0 (0.0)	7 (31.8)	2 (9.5)	26.353**(0.002)**	10 (16.7)	2 (5.1)	8.832**(0.032)**	3 (5.4)	9 (20.9)	11.065 **(0.011)**
	**2**	11 (28.9)	0 (0.0)	8 (36.4)	7 (33.3)		19 (31.7)	7 (17.9)		11 (19.6)	15 (34.9)
	**3**	23 (60.5)	18 (100)	6 (27.3)	12 (57.1)		29 (48.3)	30 (76.9)		41 (73.2)	18 (41.9)
Equipped you to identify women who are at high risk of developing perinatal mental health difficulties?	**1**	3 (7.9)	0 (0.0)	5 (22.7)	3 (14.3)	23.211**(0.006)**	8 (13.3)	3 (7.7)	8.553**(0.036)**	3 (5.4)	8 (18.6)	10.641 **(0.014)**
	**2**	11 (28.9)	0 (0.0)	10 (45.5)	6 (28.6)		21 (35)	6 (15.4)		11 (19.6)	16 (37.2)
	**3**	23 (60.5)	18 (100)	2 (27.3)	12 (57.1)		29 (48.3)	30 (76.9)		41 (73.2)	18 (41.9)
Helped you with your decision-making about whether or not a woman requires referral to secondary mental health services?	**1**	4 (10.5)	0 (0.0)	6 (27.3)	3 (14.3)	28.448 **(0.001)**	10 (16.7)	3 (7.7)	10.076**(0.018)**	4 (7.1)	9 (20.9)	12.636 **(0.005)**
	**2**	13 (34.2)	0 (0.0)	11 (50)	8 (38.1)	24 (40)	8 (20.5)	13 (23.2)		19 (44.2)
	**3**	20 (52.6)	18 (100)	4 (18.2)	10 (47.6)	24 (40)	28 (71.8)	38 (67.9)		14 (32.6)

1, not at all; 2, somewhat; 3, very well; bold type, statistically significant result; (), % of respondents.

**Table 9:** Training/education received by area and professional group.

Where have you received training/education in PNMH?		Blue area		Green area		*X*^2^ (*p*-value)	MW *n* 60 (%)	HV *n* 39 (%)	*X*^2^ (*p*-value)	Blue area *n* 56 (%)	Green area *n* 43 (%)	*X*^2^ (*p*-value)
		MW *n* 38 (%)	HV *n* 18 (%)	MW *n* 22 (%)	HV *n* 21 (%)							
Part of professional training, *n* (%)	Yes	32 (84.2)	17 (94.4)	15 (68.2)	20 (95.2)	7.818 (0.050)	47 (78.3)	37 (94.9)	5.029**(0.025)**	49 (87.5)	35 (81.4)	0.705 (0.401)
Self-directed, *n* (%)	Yes	21 (55.3)	6 (33.3)	15 (68.2)	10 (47.6)	5.138 (0.162)	36 (60.0)	16 (41)	3.413 (0.065)	27 (48.2)	25 (58.1)	0.961 (0.327)
In-practice training by a PNMH specialist, *n* (%)	Yes	21 (55.3)	17 (94.4)	8 (36.4)	4 (19)	24.320**(0.000)**	29 (48.3)	21 (53.8)	0.287 (0.592)	38 (67.9)	12 (27.9)	15.530**(0.000)**
In-practice training *not* by a PNMH specialist, *n* (%)	Yes	3 (7.9)	1 (5.6)	6 (27.3)	11 (52.4)	19.363**(0.000)**	9 (15.0)	12 (30.8)	3.517 (0.061)	4 (7.1)	17 (39.5)	(0.000)*

*, Fisher’s exact test; (), % of respondents; bold type, statistically significant result.

#### Referral pathway

Screening is integral to the referral process and this section includes data related to screening and referral practices. HVs across both areas (64.1%) were significantly more likely to always use a screening tool to assess women’s PNMH compared to MWs (21.7%), and 35.0% of MWs reported never using a screening tool compared to none of the HVs (Table 10). There was no significant difference overall between areas. Most respondents reported that lack of confidence in the results of a screening tool was not a major barrier to them referring women with PNMH problems (Table 11) but MWs, particularly those in Area 1, were significantly more likely to report a lack of time to use a screening tool as a major barrier to referring women with PNMH problems than HVs (46.7% of MWs compared to 15.4% of HVs).

**Table 10:** Reported use of screening tools by professional group and area.

Do you use a screening tool?	Area 1		Area 2		*X*^2^ (*p*-value)	MW *n* 60 (%)	HV *n* 39 (%)	*X*^2^ (*p*-value)	Area 1 *n* 56 (%)	Area 2 *n* 43 (%)	*X*^2^ (*p*-value)
	MW *n* 38 (%)	HV *n* 18 (%)	MW *n* 22 (%)	HV *n* 21 (%)							
Yes, always	11 (28.9)	14 (77.8)	2 (9.1)	11 (52.4)	36.343**(0.000)**	13 (21.7)	25 (64.1)	25.063**(0.000)**	25 (44.6)	13 (30.2)	2.962 (0.227)
Yes, sometimes	18 (47.4)	4 (22.2)	8 (36.4)	10 (47.6)		26 (43.3)	14 (35.9)		22 (39.3)	18 (41.9)	
No, never	9 (23.7)	0 (0.0)	12 (54.5)	0 (0.0)		21 (35.0)	0 (0.0)		9 (16.1)	12 (27.9)	

Bold type, statistically significant result; (), % of respondents.

**Table 11:** Reported barriers to referring women with PNMH difficulties by area and professional group.

How much of a barrier do you consider the following to referring women with PNMH difficulties?		Area 1		Area 2		*X*^2^ (*p*-value)	MW *n* 60 (%)	HV *n* 39 (%)	*X*^2^ (*p*-value)	Area 1 *n* 56 (%)	Area 2 *n* 43 (%)	*X*^2^ (*p*-value)
		MW *n* 38 (%)	HV *n* 18 (%)	MW *n* 22 (%)	HV *n* 21 (%)							
Lack of confidence in the results of a screening tool, *n* (%)	**0**	28 (73.7)	16 (88.9)	16 (72.7)	17 (81.0)	2.247 (0.523)	44 (73.3)	33 (84.6)	1.741 (0.187)	44 (78.6)	33 (76.7)	0.047 (0.828)
	**1**	10 (26.3)	2 (11.1)	6 (27.3)	4 (19.0)	16 (26.7)	6 (15.4)	12 (21.4)		10 (23.3)		
Lack of time to use a screening tool, *n* (%)	**0**	17 (44.7)	15 (83.3)	15 (68.2)	18 (85.7)	14.041 **(0.003)**	32 (53.3)	33 (84.6)	10.258**(0.001)**	32 (57.1)	33 (76.7)	4.144**(0.042)**
	**1**	21 (55.3)	3 (16.7)	7 (31.8)	3 (14.3)	28 (46.7)	6 (15.4)	24 (42.9)		10 (23.3)		
Lack of secondary care available for women who require referral, *n* (%)	**0**	15 (39.5)	12 (66.7)	4 (18.2)	6 (28.6)	10.828 **(0.013)**	19 (31.7)	18 (46.2)	2.119 (0.145)	27 (48.2)	10 (23.3)	6.473**(0.011)**
	**1**	23 (60.5)	6 (33.3)	18 (81.8)	15 (71.4)	41 (68.3)	21 (53.8)	29 (51.8)	33 (76.7)			
Lack of knowledge of referral pathway when referring women with moderately severe mental health difficulties, e.g., moderate depressive illness or anxiety states, *n* (%)	**0**	22 (57.9)	16 (88.9)	11 (50.0)	20 (95.2)	16.173 **(0.001)**	33 (55)	36 (92.3)	(0.000)*	38 (67.9)	31 (72.1)	0.207 (0.649)
	**1**	16 (42.1)	2 (11.1)	11 (50.0)	1 (4.5)	27 (45)	3 (7.7)	18 (32.1)		12 (27.9)		
Lack of knowledge of referral pathway to refer women who are currently well but at high risk of becoming unwell, e.g., ‘Red Flags’ such as women with previous history of PP/BPD, *n* (%)	**0**	25 (65.8)	14 (77.8)	11 (50.0)	16 (76.2)	4.62 (0.202)	36 (60)	30 (76.9)	3.046 (0.081)	39 (69.6)	27 (62.8)	0.514 (0.473)
	**1**	13 (34.2)	4 (22.2)	11 (50.0)	5 (23.8)	24 (40)	9 (23.1)	17 (30.4)		16 (37.2)		
Lack of knowledge of referral pathway when referring women with severe mental ill-health who you suspect requires admission, *n* (%)	**0**	22 (57.9)	15 (83.3)	12 (54.5)	17 (81.0)	6.949 (0.074)	34 (36.7)	32 (82.1)	6.854**(0.009)**	37 (66.1)	29 (67.4)	0.021 (0.886)
	**1**	16 (42.1)	3 (16.7)	10 (45.5)	4 (19.0)		26 (43.3)	7 (17.9)		9 (33.9)	14 (32.6)	

0, not a major barrier; 1, a major barrier; PP, postpartum psychosis; BPD, bipolar disorder; *, Fisher’s exact test; bold type, statistically significant result.

Unsurprisingly, respondents in Area 2 were significantly more likely to report a lack of secondary care as a major barrier to referring women with PNMH problems compared to respondents in Area 1 (Area 2: 76.7% vs. Area 1: 51.8%). Significantly fewer HVs reported a lack of knowledge of referral pathways across a range of referral scenarios, including referring women with moderately severe mental ill-health (HVs: 7.7% vs. MWs: 45%) and referring women with severe mental ill-health who may require admission (HVs: 17.9% vs. MWs: 43.3%), as a major barrier compared to MWs. There were no statistically significant differences between respondents in Area 1 and Area 2 regarding knowledge of referral pathways.

**Figure 2:**
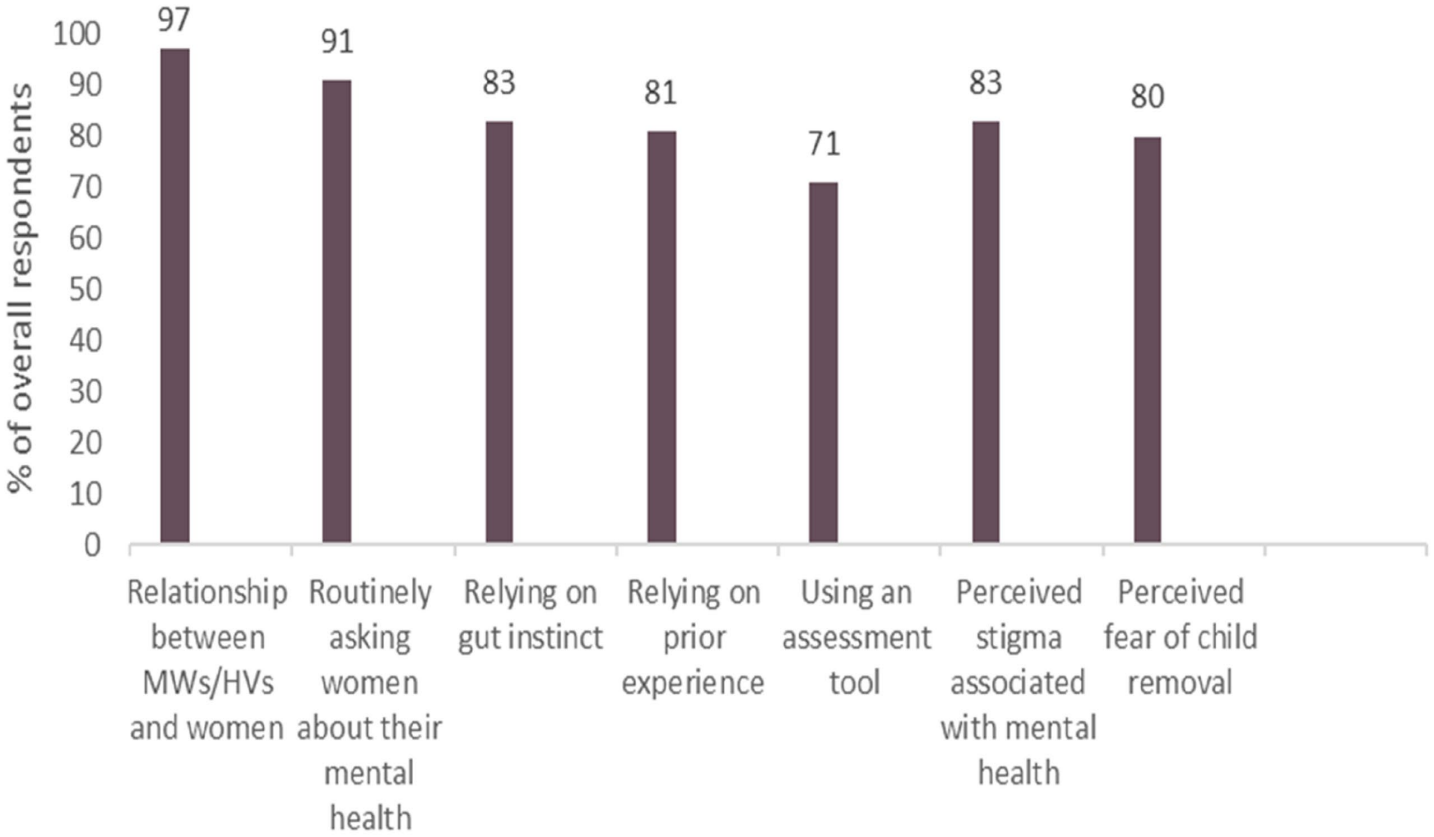
Most commonly reported perceived barriers/facilitators by MWs and HVs to decision-making about referring women for PNMH care.

Figure 2 summarises the most commonly reported factors by MWs and HVs overall that were perceived as major barriers/facilitators to making decisions about referring women for PNMH care.

## Discussion

The aim of this mixed methods research was to gain an understanding of PNMH referral decisions among MWs and HVs in the UK. Central to this was to explore any impact secondary PNMH service provision had on MWs and HVs when making PNMH referral decisions. The findings revealed that in the main participants from both areas reported similar barriers and facilitators to clinical decision-making regarding referring women for secondary PNMH care. The most reported finding identified in this research was the importance of the relationships with women. This relationship was perceived by the MWs and HVs to be vital in two ways. Firstly, a trusted relationship facilitated women’s disclosure of PNMH problems. Secondly, aided by CoC, MWs and HVs could get to know women in their care and subsequently allowed them to observe changes in behaviour and/or presentation potentially indicating emergence or worsening of PNMH problems. This in turn facilitated MWs/HVs in their decision-making about referral to secondary PNMH care. Previous research reinforces the value of a trusted relationship and echoes that this increases the likelihood of MWs identifying PNMH problems (22, 26). A trusted relationship is central to the philosophy of midwifery and health visiting care and established in the NMC midwife and HV proficiencies, respectively (31, 32). In line with previous research, a trusted relationship that develops through CoC encourages women to be forthcoming in disclosing mental health concerns (33). Although in the current study CoC was valued by both MWs and HVs, several factors limited the provision of this and therefore were barriers to building relationships and consequently to referring women for secondary PNMH care.

Across the sample, over 60% perceived reduced number of overall contacts were a barrier to identifying women with PNMH problems. Moreover, the quantitative data showed almost 75% of the HVs in both areas perceived a lack of contacts in the home was a major barrier to identifying women’s PNMH problems. Interview data suggested that HVs felt home contacts facilitated the building of relationships, and that the home environment was perceived to engender feelings of security and confidence for women thereby facilitating disclosure of pre-existing/current PNMH problems, these findings are reinforced in the literature (34). Home visits were also perceived to be beneficial to HVs as they fostered a holistic approach to their assessment of women, enabling HVs to observe women and assess how they were coping and/or observe women’s parenting. Observing women’s parenting in their own environment supports a holistic approach to care and also consider the long-term impact of mental ill-health on infants (35).

Stigma associated with perinatal mental ill-health has been extensively documented in the literature (36–39). Findings from the qualitative and quantitative phases of this study indicate this stigma was perceived and experienced as a barrier to disclosure for MWs and HVs working in Areas 1 and 2. Many interview participants stated they perceived this stigma and perceived fear of child removal prevented women from disclosing PNMH problems. This finding was reinforced in the questionnaire results as approximately 80% of all MWs and HVs perceived this as a major barrier to disclosure. Previous research supports the notion that fear of child removal is a barrier to women in disclosing PNMH problems (33, 40). Arguably, stigma may deter some women from ever disclosing PNMH problems and perhaps helps explain why a reported 50% of women do not receive the treatment they require (18) as some women may choose not to disclose PNMH problems even when directly asked by their HCP.

A significant finding from the questionnaire revealed that almost half of all MWs in this study perceived physical health checks were prioritised allowing them little or no time to complete a mental health assessment. This finding has previously been evidenced in the literature (22, 38). Notably, during interview discussions around barriers to decision-making to refer women for PNMH care, many MWs recited the list of physical health checks required at each contact, often in a short amount of time. This list was considerable and also included lengthy documentation processes. The pervasive view amongst interview participants was that there was no parity of esteem despite the evidence that suicide is a leading cause of maternal deaths (41), and that depression and anxiety disorders are the most prevalent health problems in the perinatal period (4). The lack of parity of esteem among some MWs was uncovered in the qualitative phase of this research and confirmed in the subsequent quantitative phase. Given that MWs provide care for women and their infants spanning the childbearing continuum, prioritising physical health assessments over mental health assessments could be potentially damaging for women. For future research it would be interesting to explore this aspect further. Establishing potential beliefs and attitudes about the perceived importance/lack of importance of mental health in the perinatal period would be beneficial in informing midwifery education programmes and challenge the notion that mental ill-health and its sequelae are less important than physical health. It may also provide evidence to support more time being allocated during contacts to allow for necessary physical and mental health and wellbeing assessments.

Findings from phase 1 and 2 revealed there was consistency in the tools used where the majority of participants used the tools recommended by NICE (11). Interestingly, this research supports previous literature which suggests that MWs and HVs used validated screening tools inconsistently and incorrectly, where tools such as the Whooley questions and GAD-2 are applied in ways that may affect their psychometric properties, i.e., they are asked in a conversational manner and not verbatim as intended (42). Between the two professional groups, HVs were more likely to use screening tools in clinical practice compared to MWs and this finding is in line with previous research (43–45). Qualitative and quantitative data revealed that HVs reported routinely using screening tools to assess women’s mental health whereas 35% of MWs surveyed reported never using screening tools.

Across both areas, many interview participants voiced a desire for clearer referral pathways to inform and guide clinical decision-making to refer women for PNMH care. Moreover, quantitative data revealed that approximately one third of all respondents across both areas reported a lack of knowledge of referral pathways as a major barrier when making decisions about referring women for PNMH care across a range of scenarios, e.g., women at high risk of mental ill-health problems, women with moderately severe and severe mental ill-health problems. It could be argued that this reported lack of knowledge of referral pathways were a result of an unclear pathway in the first instance as indicated by many interview participants. This finding is in line with previous research which found that MWs and HVs lack clear policies and guidelines to manage women’s mental health needs (37, 39, 46) and suggests that the lack of clarity in policy direction regarding PNMH referrals is possibly widespread and not confined to the geographical areas covered in this research.

Unsurprisingly, over three-quarters of Area 2 participants perceived the lack of specialist secondary PNMH care was a major barrier to referring women with PNMH problems. Interview data from Area 2 participants demonstrated their frustration where they had limited referral options (pre-PNMH CMHT provision) such as, the GP, signposting/self-help strategies or 999 in an emergency. Some Area 2 participants likened identifying a woman’s PNMH problems to “opening Pandora’s Box” where this led to further issues such as where to refer the woman once a problem had been identified.

Interview data, questionnaire results and open text comments revealed that routine enquiry of women’s mental health was perceived as a major facilitator for disclosure and identifying PNMH problems. However, herein presents a contradiction with the data. Whilst most respondents (91%) reported that routinely asking women about their mental health was perceived to be a facilitator for disclosure, almost half of all MWs and 15% of HVs reported that using a screening tool was not very important in facilitating identification of PNMH problems, and over a third of MWs completing the questionnaire reported never using a screening tool. This has significance for clinical practice where different methods/approaches are used by MWs and HVs to enquire about women’s mental health instead of using validated screening tools. Inconsistent approaches may lead to ambiguity whether referral requirements to PNMH services are necessary. Thus, it was not clear from the data what form of enquiry took place amongst participants who did not report screening tools as important and/or use screening tools in practice. However, it could be that the MWs and HVs relied on experience and their intuition to guide what they considered to be their routine questioning around PNMH concerns.

Intuitive practice was found to be highly regarded in this study and in the literature amongst HCPs and is an important factor when making clinical decisions (47–49). However, some authors have highlighted the subjective nature of intuitive decision-making since it is both difficult to define and quantify (50). Intuitive decisions were conveyed by the MWs and HVs in this study as “gut feelings” and “common sense” and not substantiated by any formal processes that provide evidence-based methods of assessment such as results from screening tools. Where intuition was used to guide decision-making, participants utilised other measures to aid this process such as observation and in the case of HVs, assessing women’s behaviour in their homes and/or home conditions as signs of women’s ability to cope with their baby as potential indicators of low mood/mental health problems. In the current study, intuition often took precedence over formal methods of assessment such as using validated screening tools when making clinical decisions.

## Limitations

It is important to consider the findings of this research in the light of several limitations. Two areas were selected for participation because one area provided NICE recommended PNMH services, and the other area did not provide any dedicated PNMH services to explore whether PNMH service provision impacted on referral decision-making. However, following collection of the interview data (Phase 1) and prior to conducting the questionnaire study (Phase 2), Area 2 obtained a PNMH CMHT. Consequently, responses from Phase 2 did not provide comparisons between two areas that provided diametrically opposed PNMH services. Nevertheless, Area 2’s PNMH provision did not meet NICE recommended services for women and was in its infancy when Phase 2 was conducted. Therefore, Phase 2 was still able to examine the similarities and differences between the two areas.

The questionnaire response rate of 13.1% is acknowledged as a limitation. Responder bias is a potential influence here and as such the generalisability of the quantitative findings is limited. Despite efforts to recruit a whole population of MWs, only a small number of community based MWs were recruited into this research during both the interview and questionnaire phases. Although recruitment was equally aimed at both hospital and community-based MWs, participation was unbalanced and favoured hospital-based MWs. Finally, this research has explored PNMH referral decision-making from MWs’ and HVs’ perspective. Although it did not set out to, a limitation of this study is not capturing the experiences of women with mental health problems and their role in the decision-making process when referring women for secondary PNMH care. It is acknowledged that the experiences of women and their families are underrepresented in the literature and research on this subject is of vital importance.

## Conclusion

A key finding identified from this research was that fundamental to MWs’ and HVs’ decision-making when referring women for PNMH care was the relationship between themselves and women which was impacted on the ability to provide CoC. This research suggests that the provision of PNMH services within the participating Trusts does not have a primary influence on MWs and HVs when deciding whether or not to refer women for PNMH care. Facilitators for MWs and HVs when deciding to refer women for PNMH care included CoC, routine enquiry of women’s mental health and relying on intuition. Barriers to referral decision-making include stigma associated with mental health and lack of referral pathways for practitioners. Based on the findings from this study, the following recommendations are offered for MWs/HVs to facilitate PNMH clinical referral decision-making and future research.

- Where CoC models are in place, MWs and HVs should continue to seek ways of working that facilitate and encourage the development of a trusted relationship between themselves and women.
- Once trained in their use and administration, MWs and HVs should use validated screening tool(s) to support a consistent and more reliable assessment of women’s mental health. Using evidence-based screening tools may substantiate the more intuitive assessments made by MWs and HVs to aid clinical decision-making.
- NHS Trusts and academic education institutions should provide comprehensive PNMH training (pre- and post-registration) for MWs and HVs to ensure they have the necessary skills to equip them to identify, assess and refer women with PNMH problems. This research supports the delivery of annual PNMH training by a PNMH specialist MW/HV and *via* a face-to-face method.

## Data availability statement

The original contributions presented in the study are included in the article/Supplementary material, further inquiries can be directed to the corresponding author.

## Ethics statement

The studies involving human participants were reviewed and approved by the University of Worcester Health, Life & Environmental Sciences Research Ethics Panel (SH17180018-R) and approval to conduct the research in the NHS from the Health Research Authority (HRA) (235568). The patients/participants provided their written informed consent to participate in this study. Written informed consent was obtained from the individual(s) for the publication of any potentially identifiable images or data included in this article.

## Author contributions

JJ, LJ, EB, and LH contributed to the design of the study. JJ conducted qualitative and quantitative phases of the research and wrote first draft of manuscript. LJ, EB, and LH supervised the data collection and performed critical review of the manuscript for intellectual content. JJ and EB conducted thematic analysis. JJ and LJ performed statistical analysis. All authors contributed to the article and approved the submitted version.

## Funding

The University of Worcester and Midlands Partnership NHS Foundation Trust co-funded the PhD through which this research was carried out.

## Conflict of interest

The authors declare that the research was conducted in the absence of any commercial or financial relationships that could be construed as a potential conflict of interest.

## Publisher’s note

All claims expressed in this article are solely those of the authors and do not necessarily represent those of their affiliated organizations, or those of the publisher, the editors and the reviewers. Any product that may be evaluated in this article, or claim that may be made by its manufacturer, is not guaranteed or endorsed by the publisher.

## Supplementary material

The Supplementary material for this article can be found online at: https://www.frontiersin.org/articles/10.3389/fpsyt.2023.1056987/full#supplementary-material
