# Supplementary material for: A mixed methods study to understand perinatal mental healthcare referral decisions among midwives and health visitors in the UK
Source: Front Psychiatry. 2023 Jun 12;14:1056987. doi: 10.3389/fpsyt.2023.1056987 (PMC10291319; doi:10.3389/fpsyt.2023.1056987)
Supplement: Supplementary file 1 [file Table_1.DOCX]

| **Theme** | Subtheme | Example Quotation and Participant ID |
| --- | --- | --- |
| **Identifying Need**  **Identifying Need**  **Identifying Need**  **Identifying Need**  **Identifying Need**  **Identifying Need**  **Identifying need**  **Identifying need**  **Identifying need** | Continuity of carer  Continuity of Carer  Continuity of Carer | *“I think it’s down to continuity of carer and the quality of care because it can be very difficult to pick it up. You know, you need that length of time sometimes, to actually be with someone and see them rather than you going into a house, flying in and flying back out again”.* (Midwife 4)  ******  *“I think, continuity if it works and they roll it out as they say, that would be amazing to have a midwife antenatally, intrapartum and postnatally. So if there’s any birth trauma or mental health issues from that then that midwife’s going to know and will be able to follow it through, and not have to ask those difficult questions coz she’ll have witnessed it*” (Midwife 13)  ******  *“We are very, very good, and I’ve only recently realized how good, we are very good at continuity of carer antenally and postnatally. So although we haven’t cracked the intrapartum bit, our continuity of care is very, very, good. And so it does mean that women are under a very small team of midwives so they tend to get to know their team of midwives...”* (Midwife 4)  ******  *“We used to have a model where you would generally book your women that were going to be on your GP service care.... A lot has been taken away, so that there might be a midwife who is just a booking midwife for example. She books women from all sorts of areas during her day. So she doesn't necessarily have that link with women and I think that some of that* [continuity of carer] *has been lost. So you don't get to see, you know, your booking midwife might identify it* [PNMH problem] *but then actually you then wouldn’t see them until they're 16 weeks pregnant, and that’s quite some time after their booking appointment. So you’ve not developed that relationship with them initially”* (Midwife 10)  ******  *“I talk and erm … its one of the reasons why continuity of carer is good* [to have] *as welal”* (Midwife 16)  ******  *“I’m all about the relationship. All about it… How on earth can you expect someone to come to you* [and say] *‘I feel rubbish.. or this is happening to me’ without building up that relationship I will never know. So that is the big thing for me and its very difficult for health visitors to pick these initial concerns up erm ...without that”* (Health Visitor 6)  ******  *“..you’ve seen that woman for seven, eight months antenatally, and then a couple of weeks postnatally, so you can see how their mood is and how they deteriorate, how they feel emotionally or whether they feel like they are getting support, and the ladies who do need support”* (Midwife 12)  *“Continuity of midwife. So building up that relationship for a woman wishing to disclose [PNMH problems] or/and a midwife being able to observe any changes in that woman’s behaviour. If she’s not seeing her every antenatal appointment or through to postnatally, if it’s a different midwife, they’re not going to notice those subtle changes potentially”* (Midwife 13)  ********  *“At the moment with these key contacts that we’re allowed to do …. a lot of the time it’s the nursery nurses that do the nine-month and two-year check. We’re moving from this service and its so upsetting. We can do home visits but only if there’s a need. But I think the people that have the need, are not going to come to clinic. We're not going to pick up all these cues at home or be able to open a conversation”* (Health Visitor 3)  ******  *“..the 12 month check, that was the health visitor and I believe the nursery nurses are going to be allowed to do that now and the 2 years* [check]*, the nursery nurses do that”* (Health Visitor 7)  ******  *“I know people don’t like change and we don’t like the fact that we’re not visiting, and it isn’t because we don’t like change, but you can see the difference in your practice. And I’ve not been qualified very long, erm, you know, but four years ago when I qualified, it was a completely different role to what I am doing now. You pick up more, you were able to contain people, whereby now you’re not able to. I did a 6 week review last week and you know, mum was crying on me and there was just lots of stuff that I did not expect because I’d been in and done the one visit and we were ok, erm so yeah, it throws you, it really upsets you”.* (Health Visitor 6)  ******  *“…with the more significant MH issues, I think its got to help to see somebody that you know, somebody you can build a relationship up with. Erm, because those women who have issues with anxiety or even, you know, depression, they’ve got to feel that they can trust you in order to be honest and tell you exactly how they feel”* (Health Visitor 6)  ******  *“*[lack of] *Continuity and relationships is one of the biggest bug bears that I hear talked about with my colleagues”* (Health Visitor 5)  ********  *“…but by changing into big teams like that, things are going to get even more missed than they do now. I don’t think the continuity is going to be as good” (Midwife 14)*  ********  *“..obviously there’s loads of changes going on with maternity services isn’t there…” (Midwife 10)*  ********  *“I suppose now we don’t really build up that rapport with the woman coz we don’t see them so often, so it’s difficult to get a true picture of what’s going on and maybe for her to be open and honest with us erm, because we haven’t got the rapport that we used to” (Health Visitor 7)*  *“And what I feel we’re lacking is that we don’t have those contacts anymore to really get to know them [the women] so that they feel they can tell us [about their PNMH problems]” (Health Visitor 2)*  ********  *“I think health visiting needed tightening up a bit to make it more … to sort of turn it around. You know a lot of it is now is putting the responsibility on the parent. You know, now its been over twelve months [since the change in service delivery] I can see how that works...the model is very much based on women, you know people, taking responsibility for their own health and the health of their family, so you know, not relying solely on the health visitor to do everything to, you know, take responsibility. You know, come and see us if you have a problem, you’ve got our numbers, you’ve got our clinics, come and see us… erm … asking for help is a sign of strength. You know it’s that sort of responsibility. And you know not sort of sitting around waiting for someone to come and knock on your door for a visit and telling them things. You know take responsibility...” (Health Visitor 8)* |
|  | Disclosure  Disclosure  Disclosure  Disclosure | *“I’m a firm believer that clients tell you want they want you to hear, no matter how open* [you are]*, how ‘You can tell me anything’ you are, you are going to get those who feel that they can’t say that to you* [disclose PNMH problems]. Health Visitor 4  ******  *“There are ones that will* [disclose PNMH problems]. *But there are just as many out there who never will* [disclose]*….”* (Health Visitor 2)  ******  *“It's difficult sometimes to say how many* [women with PNMH problems] *I see because sometimes women don't tell you...”* (Midwife 6)  ******  *“..because those women who have issues with anxiety or even, you know, depression, they’ve got to feel that they can trust you in order to be honest and tell you exactly how they feel” (Health Visitor 6)*  ******  *“So it all depends whether the lady is willing to tell you the information isn't it?”* (Midwife 5)  ******  *“I think women are now able to disclose and discuss openly with someone they perhaps trust a little bit more...”* (Midwife 11)  ******  *“I think there is, erm there is still a stigma attached to it [mental health problems]”* (Midwife 10)  ******  *“I believe there’s a huge stigma with mental health. Huge, huge, huge stigma…”* (Health Visitor 4)  ******  *“..people thinking mental health is still something to be ashamed of. I’d say that’s the biggest barrier”* (Health Visitor 8)  ******  *“So how can we expect women to you know, disclose it* [PNMH problems]*, if you can’t even talk about it and get supported by your own that you work with? And it’s supposed to be a caring profession …if we can’t recognise, sympathise within ourselves and our colleagues, how can we* [sympathise] *with the women?”* (Midwife 14)  ******  “‘Oh Lisa’s off sick’  ‘*What’s she off sick with?’*  ‘Stress’  *‘What do you mean stress? Why’s she stressed?’*  *It’s that attitude you know? But if you said she’s broken her leg, they’d be like ‘Oh my God’”* (Midwife 14)  ******  *“You know, even the midwives are quite happy to share their own experiences. Many of them have had babies and suffered with their mental health so that makes it more normal and allows them [the women] to talk about it” (Midwife 3)*  ********  *“And sometimes its about normalizing how she feels as well. Especially for the mild ones. And sometimes I say, actually look at the bigger picture, take a step back and look at what’s actually happening here. And just saying to them, there’s absolutely nothing wrong in feeling like this, its ok to feel this way, and what you actually need is to speak to somebody, to talk to people, erm…” (Health Visitor 6)*  ********  *“Everybody has some degree of mental health/emotional impact on becoming a parent so I would say to all the families that we see and all the mothers and sometimes the fathers as well ... actually getting them to understand their emotional health as well” (Health Visitor 4)*  ********  *“Having a baby in those first six weeks with sleep deprivation and everything like that. I know I wouldn't do it again and I've got one child and I thought I was going mad! And sometimes actually just saying to families and saying to mothers, actually you're okay to say I'm not enjoying this, you're okay to love your child and actually look at the child and go “what have we done?”. Okay, it's okay to look and often that takes the anxiety to have the health visitor go “It is hard work and it's okay to not feel great and to fake it until you can make it happen and all the rest of it”. (Health Visitor 4)*  *“Other than saying you need to go and see your GP, I would refer on [to the GP] but I wouldn't be able to go any further than that” (Midwife 8)*  ********  *“Erm so the computer system we use has a series of questions that we ask anyway when prompted. So in the last four weeks have you felt little interest in doing things, anxious, on edge or depressed or tearful. So we have those standard questions but then obviously its down to the individual midwife to deviate away from that and get a conversation going that’s a bit more meaningful”* (Midwife 13)  ******  “*We use a system* [electronic records], *which gives you prompts so you're asking the lady about how they're feeling, are they feeling depressed or feeling low or need help with their mood. So we're having those conversations with their daily checks so that starts conversations* [about mental health]..*.”* (Midwife 3)  ******  *“I'll challenge her and say well you know it isn't always easy to live with because I know, ‘coz I've had anxiety and I’ve driven my family nuts* [laughs] *I do bring myself into it in that respect”* (Midwife 9)  ******  *“ ..so now because we talk about it more and its much more of a national agenda, I would say that most women* [are] *reporting some element of either historic mental health conditions or current or altered state of mood in pregnancy…* *because we’re asking the question, women are giving us the answers”* (Midwife 11)  ******  *“I know I feel that it is increasing* [PNMH problems]. *And whether it is actually increasing or whether women are just more comfortable in talking about it I couldn’t tell you”* (Midwife 15)  ******  *“I think its* [disclosure] *getting better and I think we all talk to them* [the women] *about mental health and certainly PND is discussed fully at discharge…”*  (Midwife 3  ******)  *“There’s not enough emphasis on mental health. We’re always going on about diet and exercise and whatever, but we’re never saying you know, well actually if you’re feeling like this there might be something emotionally that we might need to talk about it and see what we can do to help you”* (Midwife16)  ******  *“We talk about the normal physiological changes in pregnancy; you talk about the sickness, the backache and constipation, those sort of things… And that’s the trouble isn’t it? People could go to the end of their pregnancy ‘Oh nobody asked me* [about my mental health]*’”* (Midwife 14)  *“I still think there’s a certain attachment of, because the big fear for women is, will they take my baby off me? … sometimes they think that goes hand in hand and the thing we do is contact social services…* [if a woman discloses PNMH problems]” (Midwife 10)  ******  *“People don’t want to admit to their friends and family and also some appear less likely to admit* [PNMH problems] *to professionals as well. Erm, through fear of failure. I think we live in a society where everything is so fast paced and so many demands on parents to be working and to be parents and keep going erm… and fear of you know, what the referral pathway is. You know, the classic ‘Oh are my children going to be taken off me?”* (Midwife 13)  ******  *“A lot of adults recently, in the last few weeks have sort of admitted to me that they think, I’ve been scared to ask for help coz you’ll get social services involved or you’d think I couldn’t cope”* (Health Visitor 8)  ******  *“I think mothers still feel we’re going to take their baby way, its that old fashioned view … that they’re not good enough mothers and the children will be removed. And we’ve got the power to do that”* (Health Visitor 7)  ******  *“Well I think that the other thing that contributes to that* [the stigma] *is that they are in the perinatal period and there’s that perception that women don’t want to be viewed negatively. And people will still, I can I tell you this without a shadow of a doubt, women will still say things to me like, ‘You’re not going to take my baby away are you?’. So a significant proportion of women, still have that fear that their child will be removed. But for all the work that we’ve done to have those discussions about parity of esteem, all that kind of stuff, there is still that belief that health visitors and the health visiting service are associated with that”* (Health Visitor 5) |
|  | Time  Time | *“Time. Time is the biggest killer for us really. We’re limited … we haven’t got sufficient staff to cope … so I can’t honestly say, I know hand on heart these women get enough time”* (Midwife 3)  ******  *“Lack of time is the biggest thing* [barrier to assessing PNMH]*”* (Health Visitor 1)  *“How busy the ward area is, that’s a major, major thing. And I do sometimes feel that you probably can't spend as much time with these ladies as what you’d ideally like. Just because there isn't … enough staff anyway”* (Midwife 8)  ******  *“I think its allowing midwives and other professionals to have more time to be with the woman, with the family, to do home visits, to be able to follow up and carry through erm, proper assessment and interviews. Erm, I think that would actually stop women from deteriorating. If they felt that they had that support at the ground level” (Midwife 12)*  ********  *“We’re quite lucky when they’re inpatients because we’ve got a supportive midwife in post and … she sees them antenatally and she’s in touch with them so there’s some continuity for those ladies. And obviously if we’re concerned on the ward we get [specialist MW] to come and see them before they get discharged. So it's quite good and better for us. And as you know working on a busy postnatal ward with 16, 17 discharges every day. Our time is quite limited with the ladies...” (Midwife 3)*  ********  *“If it is that we were concerned about a lady postnatally, [specialist MW] normally does just antenatal, but she would see a lady if we are concerned…” (Midwife 5)*  ********  *“Well the busy one [clinic] is 30 [women] and that’s in quite an affluent area and they all want to discuss something or other. Generally between 25 and 30 [women attending] is an average clinic. And that’s in an hour and a half”. (Health Visitor 7)*  ********  *We just don’t have the capacity really [to do an antenatal contact]. I sometimes think we should have, but we don't. We prioritize them, in our office, where we prioritize, we do prioritize primips and child protection, then multips might get a telephone call if we've got lots of capacity”. (Health Visitor 3)*  ********  *“So definitely, like being able to get all the post-natal checks done and all the antenatal care side of things done, make sure all the scans and everything like that. Yeah it does, it probably ends up taking away from the other parts of the job. And I think the turn over is so quick as well. Yeah. See you kind of can't sit and do that [assess mental health], which is a shame”. (Midwife 8)*  ********  *“Yeah, without a doubt [PNMH gets missed]. Coz you’ve got … let’s just say, a 28 weeker comes in. You have to start measuring her tummy, make sure they’ve got a growth chart printed off, erm 28 week bloods, you’re talking to her about whooping cough, flu vaccine, are they well in themselves, you know the health check, DV you know, you’re asking about all those things. And going through those things, sometimes I think it’s just easy to ask those things and just actually not listen to what they are saying. Because you’re too caught up because you know that after 30 minutes when that lady’s gone, you’ve got another one coming in”. (Midwife 13)*  ********  *“So you’re doing blood pressure, wee, telling them about whooping cough and everything else that goes with it. Talking about breastfeeding and talking about everything else in your few minutes. And most ladies that come into clinic are consultant care so they’ve got problems. They’re not bog standard, normal ‘OK. How are you feeling’ ‘Oh fine’. You don’t get those ‘oh fine’ ladies. You get the ones you know… so I think in clinic there’s not enough time in just a general clinic [to discuss mental health]” (Midwife 14)*  ********  *“You can’t assess someone’s mental health in that time… Oh, bloods, weights, heights, histories, everything, previous children, any problems, any concerns, yeah it’s just incredible ...screening...” (Midwife 12)* |
| **Education, Skills and Experience**  **Education, Skills and Experience**  **Education, Skills and Experience**  **Education, Skills and Experience**  **Education, Skills and Experience** | Targeting Resources | *“..our services are very stretched. We’ve got a lot less health visitors. We can’t provide the service that we could historically … we try to target the ones that are most vulnerable”. (Health Visitor 1)*  ********  *“…a woman I saw not so long ago, I had a referral from the midwife to say that she’d actually been sectioned earlier in the year erm, so I did manage to do a targeted antenatal which is unusual”. (Health Visitor 7)*  ********  *“..if I’d identified somebody who perhaps at that time had answered* [positive] *to the marker questions but really said ‘I don't really want all of the extra services and I feel like I just need to work through things’, I would just offer additional antenatal appointments, contact the health visitor about getting early access to the health visitor. But again, you know, that’s all, you know resources…”* Midwife 10  ******  *“Well I think the most important thing is the decline in the service as erm, the health visiting service and the service provided to women with poor perinatal mental health. We used to provide a very good service with the listening visits. The tools we used worked well and since we’ve stopped using the EPDS and using the universal* [questions] *we don’t capture as many women. Erm and then … so just being able to refer on and you know, a lot of women we’re missing and … which will then have a knock on effect with children’s health as well”.* (Health Visitor 7) |
|  | Confidence  Confidence  Confidence  Confidence  Confidence | *“But in fact I had one lady just recently, who gave birth to a Down's baby. She didn't know she was having a Down's baby, she’d split up with her partner during the pregnancy and came across as really coping but she was always wide eyed and I thought you know, you're not coping, you’re not coping! And one day I went there and she just cried the whole visit” (Health Visitor 2)*  ********  *“Erm I think I’d have more confidence if I knew there was somewhere to refer to. I think I would have more confidence if I knew that when I put in that referral it would happen timely, it would be quickly and well-focused and well-managed but I think dealing with mental health is like trying to knit fog sometimes. It’s tricky to get it right”*.( Midwife 15)  ******  *“yeah I do* [feel confident]. *I do, but I think that’s because I’ve been nursing a long time. And erm, I worked at* [MBU] *Unit and so had a lot of support there. And I get a lot of support from the specialist perinatal mental health - health visitor. I talk to her now and again coz sometimes I have students with me and she’ll have them with her for the day. Erm I do feel confident really...”* (Health Visitor 7)  ******  *“I will have a lot of conversations with mums* [in the hub] *in tears because of this, that and the other. And a lot of it is sleep deprivation or struggling to deal with other children, that kind of thing. And all of it is significant to that mum so it is really useful to have in that situation, some guidance really* [of supervision from specialist PNMH HV]” (Health Visitor 6)  ******  *“Sometimes you just think in your stomach that something is not quite right”.* (Midwife 3)  ******  *“… its difficult to quantify but you get a feeling that somethings not quite right”.* (Midwife 1)  ******  *“…I think I rely on my common sense … its your gut feeling at the end of the day that prompts you into doing whatever you do* [referring]*”*. (Health Visitor 6)  *“I’ve often got a sense that something isn’t right but it’s knowing the right way to open it up a little bit without opening Pandora’s Box and leaving her in a miserable mess…”* (Health Visitor 3)  ******  *“So you know, it helps you know, just talking, and your gut feeling and how you notice things like, I can remember as a student health visitor, and being in clinic and seeing a family and thinking, that family makes me feel really uncomfortable. Or seeing a mom and a baby’s interaction and thinking that makes me feel stressed, what is going on there?”* (Health Visitor 1)  ******  *“Erm I think confidence is a difficult one to assess because we’ve identified the lack of training, you always feel you could and should be doing more. But you don’t know what the more is or what it looks like”* (Midwife 11)  ******  *“… I suppose I feel as confident as I can do without the extra training. Well additional training would give me more competence without a doubt, buts that’s like anything isn’t it, you know. So with the skills I have I feel confident to know my referral pathways and who can support me.”* (Midwife 3)  ******  *“I think it’s useful to have some more training and some more, sort of scenarios of training where you know, what’s worked well and what hasn’t worked well and you know, where these services work together, you know, case studies...”* (Midwife 10)  ******  *“We don’t get much training on PNMH. I think training would help you recognize it and to see what is available to you service wise and you know what you do with that information because half of the time it's all very good saying I'm a bit worried about it and you don't really know what you've got to do with it”.* (Midwife 7)  ******  *“But it is down to education. And there’s not really any education out there is there?”* (Midwife 14)  ******  *“I think managing people with PNMH is more useful* [training needs]. *At the moment all we have is signpost knowledge, we know where to signpost to but we probably don’t have enough understanding of long term implications unless you’ve been through it yourself… I think what we really need is a focused study day around perinatal mental health that refocuses on all aspects, so it needs to be broader so we have a better understanding and how we can help the ladies. But without the resources it’s very difficult isn’t it?”* (Midwife 3)  ******  *“Erm we do get a perinatal mental health and maternal mental health erm, sort of in-house training which is quite good as a one off thing. Yeah it's a one off thing. And I think the mental health thing is so, it’s so massive… I do think that, erm I know this sounds ridiculous, but sort of like a module for health visitors completely based on mental health that would be sort of something that you would have to do once you qualified or something or be part of your training”* (Health Visitor 2)  ******  *“It would be good if there was some sort of compulsory study day or interaction I think, to make mental health education within the profession more tangible and meaningful. I don’t think its good enough using e-learning personally”.* (Midwife 13)  ******  *“I don’t think its* [PNMH] *talked about enough … I don’t think student midwives are that aware of just how common it is and how vigilant they actually need to be”* (Midwife 16)  *“… mandatory updates* [on PNMH] *but its limited. We would always look to* [specialist midwife] *for support because we haven’t got the knowledge really. We’ve got basic knowledge and signs to look for her but apart from that, that’s it”.* (Midwife 3)  ******  *“It’s* [de-briefing] *so important. But you know carrying stuff in your head and writing*…” (Health Visitor 3)  ******  *“..its not even training. Its much more of a peer support thing because other people do things in different ways and sometimes you think, that’s brilliant you know … I think sometimes just having perhaps even like a peer support group about certain things where you could have discussions about certain, erm bring up certain cases”.* (Health Visitor 2)  ******  *“So we’ve got a perinatal mental health lead and I’d go to her* [for advice]*. She’s really lovely and she’s the one that does our supervision. So I’d definitely go to her. I’d also use my colleagues”.* (Health Visitor 6)  ******  *“Its always good to have updates. So extended knowledge. So maybe new research, new thoughts, new processes, that really. The extra bits”* (Health Visitor 7)  ******  *“We can identify all we like, we can have all the education we like but in the end we haven’t got anywhere to refer people to who have the expertise who can help them*” (Health Visitor 2) |
| **Referral Pathway**  **Referral Pathways**  **Referral Pathways**  **Referral Pathways** | Use of Tools  Use of Tools  Use of Tools | “*Erm, structured tools? No, I don’t think that there are* [that the midwives use]*”.* Midwife 11  ******  *“I know there used to be the Edinburgh scale didn’t there for postnatal* [depression] *but I don’t know of any antenatal one [tool]. I’ve not seen any”* (Midwife 14)  ******  *“We just normally ask the questions about how they're feeling and normally just sort of, what we normally do in the routine postnatal checklist when we’ve just examined them, erm and its sort of just asking them how they're feeling or whether they feel like they're coping well, if they’ve got support.”* (Midwife 8)  ******  *“We use the universal PNMH scoring system”* (Health Visitor 7)  ******  *“Yes so if it’s someone that actually is very low, I will use the GAD or the PHQ”.* (Health Visitor 2)  ******  *“Erm, … well I ask the appropriate questions, the universal questions at the designated times, the universal times”* (Health Visitor 8)  *“We have our own Trust policy [for PNMH assessment tools] that’s quite clear cut really. You know we have an antenatal and postnatal mental health policy that obviously follows the Trust format. It is evidenced based and erm … is based upon the NICE guidance. And the locally commissioned service”* (Health Visitor 5)  ******  *“ We ask them … using your universal PNMH tools”* (Health Visitor 6)  ******  *“the promotional guide is very much my tool* [I use for assessing PNMH]*”* (Health Visitor 1)  ******  *“I might use the EPDS but the Edinburgh isn’t really recognised anymore I think. I’ve used the GAD as well”* (Health Visitor 3)  ******  *“So that is very much my tool* [promotional interviewing guide]*, as it encompasses everything so there’s that, and the Solihull [Approach], the Solihull is riddled through me, you know containment, reciprocity and behavior management, you know the reflective tool as well as a teaching tool. So those two would be my two underpinning things [tools] I use”* (Health Visitor 1)  ********  *“The GAD and things like that, that I would use if I had got positive answers to the Whooley questions, what professionally I was of the opinion, there's something here, I would go a bit further and put the GAD in there and actually explore it a bit deeper”* (Health Visitor 4)  ******  *“No I don’t [think the universal questions are useful]. I feel giving the mother a bit of paper actually allows her to express how she’s feeling…. the questions are quite general… the EPDS was more specific and we had a lot more positive results from that than we do from the universal* [questions]*”* (Health Visitor 7)  ******  *“The other thing I use a lot when talking about tools, is the GAD and the PHQ… and I found that was really, really good in helping mums understand why they’re feeling [anxious/low]”* (Health Visitor 1)  ******  *“Yeah I would say the tools do help me make a decision and help the woman make a decision to be honest, and help her realise I’m not where I want to be”* (Health Visitor 8)  ******  *“..I think, the universal PNMH questions would be more effective if we visited more because we would get to know the women”* (Health Visitor 6)  ******  *“it’s not just about asking those four questions, it’s about how they* [the HVs] *set their stall out. So it’s the conversation they have about mental health and well-being before they ever ask those questions. It’s about being very clear about what it is you are asking….. For me, you know, the biggest component for that universal assessment is the discussion you have about mental health and well-being and it’s about setting your stall out”* (Health Visitor 5) |
|  | Knowledge of Referral Pathways  Knowledge of Referral Pathways | *“… I’m sure there’s a pathway. There used to be but you don’t ever see it being* [used]..*”* Midwife 14  ******  *“I think a more straightforward referral pathway would be better”* (Midwife 8)  ******  *“.. the trouble is, we are in a process of change at the moment and the guidelines need updating and they need to be easier to read...”* (Midwife 9)  ******  *“..there needs to be a clear pathway of referral and follow up that’s fed back to the midwife”* (Midwife12)  ******  *“I just think the referral system could be a little bit more streamlined”* (Midwife 13)  ******  *“I think … I think the main barriers are … erm … a difficult care pathway to negotiate”* (Midwife 15)  ******  *“No I don’t think I would* [use an assessment tool]…*we’ve got a very open and easy access to erm* [specialist MW] *and I can just ring her up and say, I’ve got this lady….”.* (Midwife 1) |
