# Supplementary material for: A mixed methods study to understand perinatal mental healthcare referral decisions among midwives and health visitors in the UK
Source: Front Psychiatry. 2023 Jun 12;14:1056987. doi: 10.3389/fpsyt.2023.1056987 (PMC10291319; doi:10.3389/fpsyt.2023.1056987)
Supplement: Supplementary file 2 [file Table_2.DOCX]

**Interview guide**

*Please tell me about yourself in terms of your professional background and qualifications, etc.*

Prompts: your post, how long you have practised as a MW/HV, professional qualifications, e.g. mental health qualifications.

*How long have worked in this organisation? Do you have access to specialist PNMH services?*

*How many women do you see with PNMH problems?*

*Talk me through when and how you start to think about how a woman has PNMH problems.*

*What assessment tools, if any, do you use when assessing women’s PNMH?*

Prompts: EPDS, GAD, Whooley questions, etc.

*How and when do you use tools in your assessment?*

Prompts: Usefulness of tools, do they give you confidence in aiding your assessment of PNMH.

*What would help you with your assessment of women’s PNMH?*

Prompts: Tools, service provision, education, staffing levels, previous experience, qualifications etc.

*What guidelines influence your practice when assessing women for PNMH problems?*

Prompts: NICE guidelines, local policies/protocols.

Do you think these guidelines/policies are ‘fit for purpose’ for the assessment of women with PNMH problems?

Prompts: Do they assist you in your assessment?

*Is there anything that would assist you in your decision to refer women for secondary PNMH care?*

Prompts: Having a referral pathway, education in PNMH care/disorders, availability of services, etc.

*How do you manage mild PNMH problems?*

Prompts: What strategies do you employ, e.g., signposting women to support groups, providing extra visits, etc.

*What are the things that make it difficult when assessing women’s PNMH?*

Prompts: Confidence, experience, knowledge, workload, service provision, education, support – from colleagues, services, multidisciplinary team, etc.

*Once you have assessed a woman you are concerned about, what happens next?*

Prompts: Who do you refer, where to – MBU or not?

*What training have you received in PNMH? Is it adequate in equipping you to assess women with PNMH problems?*

Prompts: What was the training, who delivered it, is it mandatory?

*Would you like further training/guidance on managing PNMH?*

*What are the factors that influence your decision to refer women for secondary care?*

Prompts: severity of woman’s symptoms, assessment score, services available locally, woman’s individual circumstances such as family support, your previous experience/confidence in PNMH.

*When referring women for secondary care, do you experience any issues with the referrals?*

Prompts: secondary services accepting referral e.g., meeting referral criteria/capacity to accept referral, lack of confidence, professional knowledge and experience, etc.

*If you encounter problems with referrals to secondary services, what course of action do you take?*

Prompts: Do you contact in-patients’ units out of your area for availability of beds? Is this problematic?

*Do you feel confident dealing with PNMH care, in terms of referrals, management of care and follow up care.*

Prompts: What has influenced your confidence – education, previous experience, qualifications, etc.?

*If you were unsure about how to manage a woman with PNMH problems, what action would you take?*

Prompts: Where/who would you go to for advice/support? Do you rely on referral pathways or local policies to assist you in your management?

**Conclusion**

- *Is there anything I haven’t asked you that you thought I would?*
- *The key things I have learnt from this interview are:* (Clarify back with MW/HV key issues from the interview).
- *Thank-you for taking part. Reiterate assurances of confidentiality.*
